# Abortion in the United States' bible belt: organizing for power and empowerment

**DOI:** 10.1186/1742-4755-8-1

**Published:** 2011-01-05

**Authors:** Mary Ann Castle

**Affiliations:** 1 Senior Associate, Planning Alternatives for Change, P.O. Box 94859, Pasadena, California 91109, USA

## Abstract

Over the last 30 years, conservative power in the United States, financed and organized by Christian fundamentalist sects, the Catholic Church, and conservative corporate and political leadership, has become more threatening and potentially destabilizing of progressive democratic principles and practices. Powerful interlocking political, financial and social forces are arrayed against women in many Southern and Western states. They are having destructive effects on women's ability to control their fertility and maintain bodily integrity and health. Poor women and women of color are disproportionately affected by restrictions on abortion services. Strategically developed interventions must be initiated and managed at every level in these localities. It is urgent to coordinate and empower individuals, multiple organizations and communities to engender effective changes in attitudes, norms, behavior and policies that will enable women to obtain reproductive health services, including abortion care. This paper describes contextual factors that continue to decimate U.S. women's right to health and, then, describes a community organizing-social action project in a number of US' states aimed at reversing the erosion of women's right to have or not to have children.

## Part I. The Problem

### Introduction

Over the past 30 years, intensifying in the last decade, Christian fundamentalists and the Catholic Church in some United States' Southern and Western states have mobilized congregants, funds, political campaigns, and legislative and judicial support to effectively transform private religious norms and morality about sex and family structure into public law. This has resulted in an erosion of women's autonomy and reproductive rights in these states.

Religious discourse has now become a vehicle used by politicians and legislators to advance conservative ideology and morality. Charismatic evangelists preach from the pulpits of their mega-churches via television denouncing President Obama as the Anti-Christ and women who obtain an abortion as murderers or "baby killers" tantamount to felons. Such clerics and their churches also regularly violate their non-profit status by explicitly promoting political candidates by name and adopting specific political positions in their weekly sermons [1]. The controlling influence that these wealthy theocrats exert on public opinion and community social norms is an unchecked abuse of power.

Anti-female rhetoric, couched in appeals to some sort of religious morality, has become a routine component of the American political environment. Rhetoric is one thing. The reading of scripture into actual law is something quite different. In fact, the reading of scripture has become, in some states, the dominant cultural paradigm, profoundly anti-female and sustaining gender inequality.

Anti-abortion activists have developed a language of blame and shame. These accusations are based on biblical passages that form a picture of women who chose to have an abortion and their physicians as murderers.

### Interconnected systems

The Christian Right raises billions of dollars to support ultra-conservative state-level candidates and legislators to promulgate their religious views. These philanthropists have developed interconnected funding priorities and strategies to advance their public policy agenda. For example, they provide fellowships and offer professional supports to public officials. Networks of regional and state policy right-wing think-tanks and advocacy organizations have been created to provide testimony to legislators and directly influence them. Money from corporate businesses, wealthy individuals, conservative family foundations and owners of media strengthen and expand their ability to influence public policy [2].

For example, conservative foundations such as The Heritage Foundation, Focus on the Family and others, owners of large conservative corporate businesses, such as Coors Brewery, Curves for Women and several media conglomerates such as FOX television news undertake research, produce anti-abortion messages and write anti-abortion laws and policies in states across the nation [3]. They have established comprehensive and intersecting communication structures, attracting people to their ideological viewpoints through proselytizing and the effective use of popular culture. For example, the Oral Roberts Mega Church and University in Tulsa, Oklahoma recently organized a free concert for young people. Billed as "Genocide: A Night for Life," the Saturday night event propagated the idea that abortion is equal to genocide for communities of color [4]. These foundations have provided strategic financial support to Political Action Committees (PACs), such as the Republican State Leadership Committee and the Club for Growth, that lobby for legislation and ballot initiatives and support pro-life candidates for political office [5].

The corrosive influence of this material and symbolic power is demonstrated by administrators of human service organizations in many Southern and Western states, who fear that their conservative board members and philanthropic foundation supporters will destroy their agencies by withholding funds, if they offer abortion referrals or services to clients.

Another huge influence has been the Catholic Church which now owns many health and social service institutions throughout the United States. It has threatened agencies with the loss of funding, if they offer information or referrals to abortion care to clients. Religious restrictions in secular and state run hospitals that have merged with or are managed by Catholic hospitals are interfering with the doctor-patient relationship. Across the nation, physicians in these hospitals have been prevented from using standard medical treatments, even in cases of emergency, such as treatment of ectopic pregnancy with a drug also used to induce abortion or routinely offering emergency contraception to rape victims [6]. Poor women and women of color are disproportionately affected by these restrictions. Wealthy women can and have left their states to secure an abortion.

### Legal

The intertwined systems of wealthy religious fundamentalists, conservative philanthropic organizations and corporate magnates have successfully built political alliances that have resulted in federal and state laws and policies that restrict access to abortion. Approximately 15-20 legal restrictions have recently been enacted by conservative state legislators that seriously curtail access to abortion. States with the most serious restrictions include: Arkansas, Idaho, Kentucky, Mississippi, Missouri, Oklahoma, North Dakota, South Dakota, South Carolina, Utah, West Virginia and Wyoming These laws include the following restrictions:

(1) Limits on the number of abortions a physician can perform;

(2) Requirements for abortion facilities to have operating rooms/surgical centers to perform both medication (the pill)and surgical abortion;

(3) Forced biased counseling and mandatory delays (24 hour delays, counseling bans and gag rules, and policies preventing public hospitals and public employees from performing or counseling women about abortion);

(4) Permission is granted to health care providers and entire facilities to refuse to provide abortion services,

(5) Refusal to allow Medicaid (federal health insurance) to cover abortion, thereby, restricting low income women's access to abortion;

(6)Requirement of parental consent for young women to have an abortion; and

(7) Laws stating that only physicians may perform abortions, preventing advanced practice health professionals from offering this medical service. Nurse midwives, nurse practitioners, and physician assistants are often trained in and utilize the same techniques in other procedures that are needed to perform an abortion. Yet, these specialists are prohibited by law from performing this medical service in these states.

A recent tactic by conservative legislators has been the introduction of fetal personhood laws that confer the status of personhood to the fetus and unborn child, making it the victim and ignoring the mental and physical suffering of the mother. Legislators in North Dakota, Montana, Maryland, Alabama, and South Carolina have already introduced bills.

Oklahoma is in the forefront of instituting legal measures to suspend women's civil liberties.

Recently, Oklahoma legislators passed a law requiring all women to undergo an ultrasound test and be shown the monitor as they listen to a detailed description of the fetus one hour before having an abortion [7]. The law states that the "clearest technology" must be used in this test, which is a vaginal ultrasound. Although other states have passed similar ultrasound measures, Oklahoma's law goes further. It mandates that a doctor or technician set up the monitor so the woman can see it and to describe the heart, limbs and organs of the fetus. No exceptions are made for rape and incest victims. A second measure passed into law this year prevents women who give birth to a disabled baby from suing any person who might have withheld information about fetal anomalies and which might have resulted in the woman obtaining an abortion had she been informed [8]. This law, in effect, denies women critical medical information about the health of their fetus that might cause them to seek medical interventions to improve the outcome at birth, or, conversely to abort a fetus with abnormalities that might lead to infant death or a lifetime of severe disabilities. The Oklahoma law legally indemnifies those who mislead or misinform pregnant women in an effort to impose their personal beliefs on a patient.

The Oklahoma law basically protects the numerous Crisis Pregnancy Centers (CPCs) that are funded by conservatives and the Catholic Church. There are more than 4,500 CPCs throughout the U.S. Eighty-seven percent provide medically inaccurate information by recruiting abortion clients through false advertising [9]. These "fake clinics" mostly rely on volunteer counselors with no recognized counseling or medical training. The primary purpose is to persuade teenagers and women with unplanned pregnancies to choose motherhood. Neither abortions nor referrals to abortion clinics are provided. Women describe being harassed, bullied and given blatantly false information by CPCs. Many CPCS offer free ultrasounds of the fetus to pregnant women and often do not have technicians who are trained and licensed to interpret the ultrasound.

Finally, another law that was debated, but not yet passed by the Oklahoma state legislature in 2010 would allow the woman, her spouse, her parents, siblings, guardian or current or former licensed health care provider to sue for damages, if she does not have this ultrasound [10]. If enacted, this law would de-individualize the woman, restoring something like clan ownership of her body. Through its multi-kin and health care provider intrusiveness, it would seem to imply that women are chattel or owned by the family and community. The laws discussed above are used as primary instruments to suspend women's civil liberties.

### Health care and the Government

Health care decisions are increasingly made by managers and insurers in the US with different views about abortion instead of by doctors and their patients. This bolsters anti-abortion laws and policies. A large number of HMOs, group practices, hospitals (especially Catholic Health System hospitals) and clinics have blunt "no abortion" policies. The recent federal Affordable Care Act imposes restrictions on access to abortion care and affects abortion coverage in private insurance plans in an unprecedented manner. The law excludes family planning from medical insurance [11].

### Targeting communities of color

Two fundamentalist organizations, Radiance and Bound for Life, have introduced a campaign equating the Constitutional right to an abortion with an act of genocide against women and communities of color. Huge billboard advertisements have been erected in the state of Georgia proclaiming that *"Black Children are an Endangered Species because of abortion*." This campaign is directed at African Americans, Latinos, Native Americans, and prison inmates-- the very populations that have historically suffered in the United States from forced sterilization, testing of clinical drugs without true informed consent and racist practices and policies. The idea is propagated that white organizations (e.g., Planned Parenthood, abortion clinics, pro-choice OBGYN physicians, etc.) want to prevent the birth of children of color in some horrendous genocidal plot. Attacks such as these against women's Constitutional freedom are inextricably intertwined with the long-standing American scourge of racism.

### Economic Constraints

The current economic crisis has resulted in women not being able to pay for expensive contraceptives (monthly hormonal, IUDs). Even middle class women delay abortions in order to gather funds [10]. Most private health insurance companies do not pay for abortion or contraception. Laws have been enacted that disallow low-income women who have federal health insurance (Medicaid) from having an abortion paid for by this insurance. The new federal health care reform legislation even disallows private insurance companies from paying for abortions, if they also receive any type of federal support to cover women who had not been previously insured.

### Lack of medically accurate information about reproductive and sexual health

Under the Bush Administration, $1.5 billion in tax dollars funded abstinence-only reproductive health and sex-education programs. This has been despite of the fact that research has clearly demonstrated that abstinence-only programs are ineffectual in preventing teen pregnancy [11,12]. One conservative Ohio Website that promotes Abstinence Till Marriage (ATM) for young women received $1.6 million in federal grants for "educational outreach." In 2009, this ATM website not only contained misinformation, but promoted violence against women (in essence, condoning rape) until women's rights advocates demanded that the misogynist propaganda be removed (see: http://www.psclebanon.org/atm.html).

### Violence

Anti-abortion protestors harass women clients, physicians and their staff at clinics, and at home. Threats on the lives of clinic directors have become so common that they are no longer reported in the public press. For example, in some states, Catholic school children wearing their school uniforms are brought to protest in front of abortion clinics by adults screaming invectives of "baby killer and murderer" at clients and staff. And this sort of behavior seems normal across the conservative states. Protestors routinely sneak into the clinics causing havoc. In two Southern clinics, protestors cut holes in the wall and pumped butyric acid into the clinics causing them to be closed for weeks. Operation Rescue/Operation Save America have printed WANTED posters with photos of doctors who perform abortions and have distributed them at the doctors' homes, offices and in their neighborhoods. This extremist tactic was carried out in Pensacola, Florida in the 1990s and preceded the murders of two other providers and a clinic volunteer [13]. The increasingly violent rhetoric about "baby killers" can result in real violence as seen in the recent murder in Kansas of physician George Tiller who provided abortion care. Amidst such domestic terrorism, few physicians are willing to provide abortions in these states.

Stigma and harassment about abortion leads to public humiliation of women and their physicians. Protestors routinely photograph women and staff entering clinics. Photos, captioned "murderer," are then posted on church walls or near the women's and staff members children's schools. These propagandistic attacks on women have resulted in the loss of jobs, being barred from churches, community events, etc. Fearing stigmatization and public accusations as "murderers," women (and their supporters) who have abortions have been effectively silenced. Silence, inadvertently, supports the "violent perpetrator" label encouraging greater legal restrictions on women's bodily integrity.

In some cases, states are active participants in stigmatizing women who have had an abortion. For example, Oklahoma state law requires physicians and clinics to report detailed personal information to the Department of Health that will be posted on its website. Although names are not included, women in tiny rural communities can readily be identified by this information. The religious, business and political power in some states has resulted in a hegemonic silence or neutralization where even pro-choice individuals will not openly speak or support abortion rights.

The Orwellian language of the fundamentalist and conservative forces has stigmatized and labeled women who seek abortions as "perpetrators of violence." By conferring the status of "victim" or displacing the locus of victimization to the fetus, all women who seek abortions--including "victims of rape and incest" -- are accused of being murderers. Recently, violent talk has accelerated among the fundamentalist-conservative right, such as the radio talk show host, Rush Limbaugh. Inflammatory language and behavior could be interpreted as a yearning for violent repression on the part of some fundamentalists and conservatives [14].

### Fundamentalist women and abortion

Women who are members of these fundamentalist congregations and have picketed abortion clinics have, themselves, sometimes need abortions. They have entered those very same clinics, but in disguise. The powerful structural, environmental and symbolic forces arrayed against abortion make it impossible for these women to empathize with other women. These women take on the viewpoint of those who dominate them. They suffer the shame, humiliation, anxiety, and guilt that come from accepting the beliefs of the men who provide the basis of their social identity. Their beliefs are reinforced by the relations of power and gender domination in their households, schools, churches, and communities that constitute their social universe. They must distance their own experience with abortion from that of others in order to maintain their moral self image within the narrow and bounded confines of their social universe.

### Summary

Interlocking structural (legal, economic, health services) and symbolic systems (using images of victimization, murder, violence, religious morality, etc.) reward adherence to anti-abortion and anti-female views. Deviation is punished in many Southern and Western communities throughout the United States. Growing numbers of people and organizations are complicit in defining women who have abortions as essentially the OTHER. The near universal silence about abortion in many of these states makes it appear that the anti-abortion perspective is a matter of common sense--a taken for granted reality, and that abortions happen somewhere else, perhaps outside of their state. This perception contributes to the inability to question the experience of all women's lives and their need to control their fertility.

The harassment, humiliation and stigmatization of women in need of abortions and protection of their reproductive health rights have effectively silenced individuals and organizations in many US states. The overall anti-woman strategy includes inflammatory and violent language, the strength of fundamentalist Christian religious organizations that are financed by conservative family foundations, corporate executives, and the media. Popular culture is used to disseminate ideas equating abortion with genocide. There is also the provision of medically incorrect information about sexuality and reproductive health. This is disseminated through churches and schools through government-financed and ineffective abstinence-only programs. The mass media collude with anti-women's rights talk show hosts, websites and blogs. Conservative political apparatus promotes state legislators who vote to increasingly restrict access to abortion and pass misogynist policies.

These inextricably connected systems and tactics of humiliation, harassment and stigmatization have effectively silenced even those who are personally pro-choice and, yet, cannot defy the prevailing norms of their communities, funders, religious leaders, board members, teachers, etc. about the need for abortion services as a normal part of comprehensive women's health care. Their own silence is complicit in obstructing, and more often than not, denying the right to health and personal autonomy to women in these states.

## Part II: What is to be done?

How do our understandings of the interlocking and self-sustaining forces of the Christian Right, corporate America, and political conservatives inform our efforts to increase access to abortion and return to women the rightful control over their bodies? Clearly, there is a need for a multi-dimensional and integrated approach to this complex problem. It cannot be addressed piecemeal.

To counteract hegemonic silencing, stigmatization, and well-founded fears of violence, on one hand, and the cluster of anti-abortion beliefs and state laws, on the other, an alternative approach must be tried. A strategy is needed that is based on the solidarity and collectivity of all women and the organizations that serve them. To achieve this, people and organizations must be met where they are. People's real concerns that emanate from contradictions within the social system require a thoughtful response. Simultaneously, a growing awareness of people's concerns must be nurtured and new ways to solve those problems identified [15]. Working at the intersections of multiple issues can create such change.

### A new approach

Funded by a non-profit organization in 2008, an innovative, demonstration project was initiated to increase access to abortion in several U.S. Southern and Western states. The chosen states have highly restrictive abortion laws, little grassroots organizing, marginalized populations and large rural areas. To avoid sabotage or violence by anti-abortion extremists, the names of the states involved and the funding agency will not be divulged.

The project's overarching goal was to cultivate a critical mass of diverse organizations and marginalized communities, capable and willing to re-think training, service delivery, support and advocacy strategies to promote reproductive health, including abortion services. This organizing work took place within the context of state and population-specific cultures, norms, politics, and health care systems. It aimed to build alliances between progressive organizations in each state. The relationships included the mostly small number of traditional reproductive rights activists *as well as *health and human service organizations that serve women *and *marginalized communities. None of these latter organizations and communities has been previously involved with women's reproductive rights. The project worked collaboratively with them to broaden the challenge to conservative institutions, influence, politics and norms that thrive in these states.

### The Project's Framework

The project drew upon the success of past social movements. Adhering to a model of feminist organizing, project organizers in each state created and maintained relationships, provided resources to and developmentally engaged with a diversity of service and advocacy organizations and communities that were new to issues of reproductive health and rights [16]. This empowerment process created collaborative knowledge, new initiatives, and action. Rooted in the thinking of Paolo Freire, the project embraced the idea that when people come to their own understandings of oppression, they can create ways to overcome it [17]. Each state project worked to articulate a counter-hegemonic account of the objective realities of how women and families live and how they understand those realities in relation to reproductive health and abortion service needs [18].

Deliberative discussion with new organizations and communities by project organizers encouraged recognition of the range of women's reproductive health care needs, including abortion services. Organizers also offered financial and practical assistance to agencies to develop new ways to meet these needs. Such supports provided to diverse organizations that serve women and their families enabled them to initiate or strengthen reproductive health referrals and services for their clients.

For pragmatic and political reasons, the project did not focus exclusively on abortion rights. Social control and political power described earlier have produced universal fear and intimidation in targeted states. As a consequence, organizations and individuals are afraid to risk direct involvement in actions addressing the serious lack of abortion services for their clients, constituents, and themselves. A sole focus on abortion would have seriously limited the number and type of organizations and communities with which working partnerships could be developed. The project's ability to decrease barriers and increase access to abortion would, thus, have been substantially constrained at the outset had the project had a single issue focus, i.e., only abortion. Instead, efforts were geared to shifting the discourse from demonizing women who have abortions to normalizing abortion within women's health and lives, thereby, making it *every woman's issue*.

### From theory to practice

A fundamental premise of this approach is that real and sustainable change can only occur when members of local communities directly engage with one another to strengthen access and service capacity and collectively increase their influence over political power and policies. Organizers can create these linkages among agencies and communities. The project, therefore, hired a lead organizer for each state, who had a profound knowledge of cultural differences and political nuances in their state. Each state organizer, in turn, hired and supervised other organizers with specific skills and knowledge. As a result, the locus of control in this initiative was not centered on outside "experts," but with insiders who were well known and respected within their own communities and who had authority in their local context. Organizers identified critical contradictions and recognized and seized or created opportunities that might generate collaborative possibilities. Each state's project adhered to the overall framework, goal and objectives. Yet, each operationalized the work differently based on the contextual particularities of the state.

Women and others are easy targets of social control through social, emotional and intellectual isolation. Thus, the project organizers worked with and through grass roots organizations that serve women and through networks of assistance to women. A core strategy was to engage a diversity of organizations and communities that had not been previously involved with reproductive health, but whose clients and constituents would benefit from greater access to services and care. Diverse communities and organizations were engaged in articulating and critically examining the intersections of women's health and social service needs and the need for abortion. The emphasis was on the shared experiences of women administrators, staff, clients, constituents regarding reproductive health. When experiences of health inequality are widely perceived as acute, opposition to anti-abortion norms can emerge [19]. Thus, relationship building through collaborative discussions, organizational assistance and staff development provided to these new organizations and communities developed a more coherent narrative of women's experiences and need for abortion and other reproductive health services.

This organizing approach facilitated a process that encouraged thinking about the range of unmet women's health care needs, asking questions about these needs, and, ultimately, meeting those real needs. A more favorable terrain was created to disseminate alternative ideas (i.e., those that are counter-hegemonic to the near-universal anti-abortion norms) about the range of reproductive health unmet needs, including abortion. Material support and assistance enabled agencies to develop concrete services and/or solid linkages to these services.

### Autonomy and flexibility of state organizers

The organizing approach in this initiative was conceived of as flexible and encouraged creativity by the state organizers. Different viewpoints were voiced by organizations and communities that stimulated organizers to think more deeply about and analyze situations as they arose. This helped solve emerging problems that could become barriers to effectively meeting goals and objectives [20]. There was also a collaborative advantage in that the project was implemented simultaneously in several states. Organizers shared experiences, problem solving techniques, and discussed unanticipated challenges and potentialities across states. This enabled a "natural" network of organizers to emerge. The funding agency supported the local state initiatives by providing the broader conceptual framework, creative ideas and problem solving, significant funding and fundraising assistance, substantive resources, technical assistance regarding knowledge, experience with training and supporting physicians in conservative environments, and connections to national NGO's with which local organizers could collaborate.

### Risks to organizers

The work of the organizers required courage, commitment and nimbleness. They and their families were at great personal and professional risk in engaging in this work. Should the anti-abortion systems identify them, they and their families could be harassed, potentially ostracized and possibly violently harmed. Obviously, harassment and ostracism would damage these organizers' integrity, credibility and trust within their own communities and within the organizations and communities with which they were collaborating. Professionally, they could be fired from other jobs, be black-listed and unable to secure future career opportunities. This could occur despite the fact that abortion was, but, one aspect of the organizers' work to ensure that women in their states can easily obtain the full range of reproductive health care services. To maintain morale, organizers focused on small successes as forerunners of future outcomes.

### Building a Southern strategy and tactics

Although flexible in the general approach, state organizers followed a basic tactical plan of action to address how the work should be carried out within the conceptual framework. Using a capacity-building model to engage new organizations and communities, organizers created their own networks and alliances throughout the target states. They offered information, developed skills, and generated collective knowledge as they undertook grass roots organizing and political advocacy. The approach was holistic with each activity or component supporting the other. The ultimate aim of developing networks of newly involved agencies was to build state wide organizational infrastructures.

Specific activities with communities and organizations included: (1) Provision of information, education and communication, (2) Relationship building through dialogue and critical inquiry with organizations and communities, (3) Structured collaboration (e.g., help to design a new service) to identify and carry out interventions, (4) Organizational development or capacity building, such as professional training, mentoring, resource development, creating collective knowledge, and (5) Building community capacity by creating and strengthening alliances, linkages, networks and encouraging collaboration with local progressive networks/coalitions with different issues and, ultimately, with national-level social justice organizations.

Ultimately, the work built and supported an organizational infrastructure in each state that can actively and vocally support women's right to comprehensive reproductive health care, including abortion. Local organizers in each state engaged with new community partners by using critical inquiry to enhance their understandings of the specific nature of the problem and the relationship of the organization's mission to reproductive health and abortion. They, then, collaboratively designed and implemented interventions that will, over time, improve client or constituent access to reproductive health and abortion care. In the process, strong support systems and overall commitment and capacity were developed and solidified.

### Creating alliances and building state-wide networks

The work was intended to link organizations, communities and services both horizontally and vertically: *Horizontal linkages *were between the human service agencies *and *a diversity of communities *and *reproductive health care providers. *Vertical linkages *were between the new organizations and communities and existing or new progressive coalitions at the local, state and national levels. The approach was developmental as it takes time to accomplish individual behavioral, organizational and social change, especially in extremely conservative environments with anti-abortion state laws. Initially, work entailed making presentations at professional association meetings (e.g., statewide meetings of nurses, social workers, environmentalists) or at meetings of providers of similar services (e.g., housing, jobs, youth programs). Some organizers convened small meetings of representatives of specific communities or simply one human service organization.

Usually, such presentations addressed the dearth of reproductive services in the state or county and the health consequences for women and their families. People and agencies were identified from these venues who expressed an interest in further exploring the topic. Organizers followed-up and engaged in dialogue with individuals on a one-to-one basis or in small groups, drawing the interconnections between their work and reproductive health, including access to contraception and abortion.

Organizers worked with lay and professional health care providers, human service organizations, medical education and residency programs, and agencies that offered services to marginalized communities, i.e., rural populations, low income women, African American, Native Americans, etc. those groups with the least access to reproductive health services. Examples of the types of organizations included were those that provide domestic violence support and shelter, homeless shelters, outreach to pregnant and parenting women living in remote areas; teen parenting and youth programs; and clergy. Organizers provided information and education about reproductive health care including abortion, adoption, contraception, STIs, HIV/AIDS prevention, domestic violence, and postpartum depression.

Mutual learning was the core function of the engagement period. Cultivating mutual respect between organizers' values and objectives and those of the new organizations or communities they worked with was a necessary first step. Making the importance of consequences of limited access to reproductive health care relevant to the agency's staff, their families, and clients led to a request for more in-depth information and serious reflection [21]. This approach can result in subsequent action.

Organizers and agency administrators, staff, and even some Board members listened to one another. By deepening the conversation and elucidating the connections among HIV prevention and treatment services, for example, and the need for greater access to comprehensive reproductive health care, including abortion, staff and administrators often came to their own understandings about how inequalities in these services affect themselves, their families and their clients. In the process, people became aware of their own power to change their attitudes, thinking, and behavior within and without the organizations in which they work [22].

Different communities and organizations were at different stages of readiness to reflect on, design and implement interventions to increase reproductive health services or referrals to care. With less ready agencies, discussions focused on defining a less controversial issue. For example, with an agency that was concerned with the need for women who flee sexual violence to be able to quickly and confidentially obtain emergency contraception, organizers brainstormed with staff about alternatives; and then, together, identified and implemented an agency-preferred solution.

For others, points of entry that worked for each agency, within the context of its culture, infrastructure and readiness to change were identified and concrete interventions designed.

### Examples of capacity-building

Several capacity-building efforts were used to assist partner organizations and communities to incorporate the new reproductive health services, including information and referrals to abortion care, into their missions and services.

### Professional development

Organizers worked with rural outreach staff at different locations, building upon staff's trusting relationships with clients, knowledge of cultural norms and practices, and the fact that they lived and worked in their own communities. Organizers engaged with them in learning new relevant knowledge and enhancing their communication skills with clients. Capacity-building in this way is a key to grassroots organizing [23].

Organizers also helped produce local resource guides to reproductive health care, education, and social services and assisted agencies to maintain structured referral systems to help people obtain services that they need and want. In some instances, financial support for and mentoring was provided by organizers to support a "reproductive health care team leader" within a specific agency. These team leaders were prepared to continue training new staff or retrain existing staff. This formal position helped to guarantee that reproductive health and abortion information and referrals were routinely incorporated into service delivery.

### Establishing support networks

Some organizers designed state-wide engagement and networking campaigns with other progressive coalitions, with community based organizations, human service and reproductive health care agencies and advocates. These projects were centered in some of the most culturally and geographically isolated and poorest regions of the states. The experiences young women faced in their efforts to secure reproductive health services have been documented. Case histories were used to educate various audiences through different venues about the appalling state of reproductive health in the region. Local advocates and potential advocates were recruited and trained about these issues. Eventually, they were assisted to initiate advocacy strategies aimed at securing an expansion of services. Numerous organizations have committed themselves to be part of supportive networks to ensure that women obtain the reproductive health services they need by providing transportation, childcare, funding, a safe house, etc. for women who must travel seven or more hours to an abortion clinic in their state.

### Engaging new communities

In some states, organizers provided informational workshops about reproductive health for their growing immigrant populations. Women were identified who wished to be sources of information and referrals within their communities. They also acted as personal advocates, accompanying and translating for community women. Access to contraception and abortion has enormously increased in these communities with severe political, cultural, and economic restrictions on women's agency to obtain such care. Prior to this, most new immigrants had virtually no access to abortion or other reproductive health services.

### Using technology for information, education, and referrals

Elsewhere, organizers and their partner agencies identified the need to address the near-total lack of medically accurate reproductive health information and service referrals available for youth and young adults. To accomplish this, they developed a unique collaboration with a national organization. Social media internet sites and new information technologies (twitter, etc.) were used to inform young people about the range of reproductive health issues, where and how to secure services to protect themselves against sexually transmitted diseases, and unintended and unwanted pregnancy, etc. Youth-friendly health care providers and youth development programs were an integral part of the project. Approximately, 10,000 youth and young adults living in one urban area were reached during the first year of the project. Organizers also assisted college students to research available health services at their campus health centers and engage in actions to demand comprehensive health care services. Students from these colleges were supported to attend a national activist conference. Energized, they designed a statewide young activist forum about the intersection of women's reproductive health, lgbt issues, prisoner treatment, drug policies, labor, etc.

### Working with the medical community

In some states, organizing strategies included providing technical assistance to medical students to help them learn about relevant laws and policies and to assist them to become involved in women's advocacy work. Every effort was made to identify and organize training for physicians who were interested in and willing to provide abortion care. In one state, a physician was assisted by the project to secure a large grant to routinely train her residents at an off-site abortion clinic. The physician did not have the time to write the grant. Nor did she have the experience to negotiate the training with her medical institution that is prevented by state law from training residents or providing elective abortion. Many medical residents originate in and remain within that state. They were well-trained and encouraged to provide abortions. It was anticipated that this committed physician would also be involved in the mentoring of the new reproductive health team leaders at the community agencies with staff who were trained by the project.

### What is needed to move from service provision to advocacy to construct a common pro-women identity?

The organizations that have been newly involved in providing reproductive health information, referrals or services must now be integrated into an effective coalition, if they are to successfully challenge structural, material, and symbolic forces that undermine women's right to control their fertility and their lives. And that is to integrate these groups into advocacy coalitions to ensure that alliances are not short-lived. There is a desperate need for the newly involved organizations to work together across disciplines to construct an identity built on the *collective human rights of women*. This is necessary to: (1) break the dominant cultural hegemony of silence and stigma about abortion, (2) help people to understand their commonalities with the lives of women, (3) overcome fear and threats to give critically needed political support to pro-abortion legislators, and (4) bring pressure on state and national legislators to begin to reverse the history of anti-abortion legislation.

In order to be most effective, there is also a need to work across states to share approaches that yield success and continue to provide a supportive network for organizers. Through conference calls and cross-state meetings, state organizers will need to continue to share materials and ideas and strengthen their mutual support. Some state organizers have begun to use the internet to counter the Rightist messages, to decrease social isolation and to mobilize against attacks on women's constitutional and human rights. This model suggests the benefits of seizing upon available opportunities through existing service providers to disseminate the message, deliver services, and stimulate women to think about reproductive health as part of their overall health needs. In turn, this could help all state organizers to move ahead with the larger advocacy and social justice agenda. Taken together, the work can expand health equity, cement progressive coalitions and build new alliances and constituencies.

It takes time to engender a counter hegemonic idea to the dominant anti-abortion consciousness and to develop an emergent advocacy identity within many diverse organizations or communities that is focused on women's right to control their reproduction. Without a critical mass of engaged communities and organizations, legislation will remain under the control of powerful state conservatives and their fundamentalist enablers. They will continue to disregard the reproductive health care needs of women, trample on constitutional rights, and demolish the separation between private, individual religious beliefs and government's public health responsibility.

### Creating vertical alliances

Organizers must introduce organizations to each other and create broader networks of support. For example, staff at domestic violence shelters were introduced to staff at abortion clinics or with pharmacists who stock emergency contraception. Overall organizational capacity is, thereby, strengthened to help clients gain access to needed care while transforming staff and clients' popular notions about pregnancy termination and women's right to health.

**The next step **in this type of work must be to encourage new allies to participate in coalitions or networks engaged in political action. In creating diverse, new pro-female/pro-choice constituencies, partners must be meaningfully integrated into existing or newly created progressive or reproductive health coalitions. When the newly involved agencies are ready, they must be helped to participate in progressive coalitions directed toward preventing the continued slippage of women's rights. Some organizers have worked to create reproductive health caucuses within existing coalitions that are otherwise dedicated to different issues such as: families and children, LGBT discrimination, prisoner health, worker's rights, etc. Others created new structures or coalitions where none existed. Such work entails dialogue and advocacy to reframe each distinct issue as everyone's issue. It becomes a collective tactic to confront the landscape of power within a particular place.

There may be a caveat here. Some of the literature on organizing suggests that alliances between organizations tend to be short term and emerge only from a specific triggering event [24]. To overcome this problem, organizers must work with their own states' progressive coalitions to ensure that the new organizations and community representatives have meaningful roles. They could begin by working towards cross-agency and cross-coalition meetings to build relationships in the context of working together on local health issues or by linking the local health issue to a national issue. An example would be advocacy to prevent state legislators from refusing to participate in specific measures of the health reform that would help all people in the state [25].

### Links to national organizations

State coalitions and national rights organizations can foster greater social cohesion, equality and solidarity among and between these newly engaged communities, organizations and coalitions with other progressive issues. Over time, organizers must meet with national reproductive and other rights organizations interested in and willing to work with the newly involved agencies that emerged from this work. Such conclaves can bring more organizers, greater funds, and new ideas into the target states. In so doing, the need to increase access to more high quality reproductive health services can become a priority women's health issue.

Discrimination and inequality are rarely about one form of oppression [26]. By linking newly engaged organizations and communities to diverse progressive coalitions within a state, and then by linking them with national rights groups that work to eliminate different inequalities, the projects can work towards building a broad human rights movement. Currently, elements of this movement remain fractured and lacking in cohesiveness.

### Concluding statement

In environments where powerful, wealthy conservatives use religion to influence political decision-making and shape and maintain social norms, it is necessary to develop a critical mass of individuals and diverse organizations that together can and will: (1) offer reproductive health services or referrals to providers and, eventually, (2) support advocates at the sate level. Broader constituencies can successfully demand that legislators do not vote for more restrictions and limitations of the sort that have decimated reproductive justice and health equality. This can be accomplished by restructuring, expanding and shifting balances of power in these states.

The approach described in this paper takes a cumulative and long term view of power and of organizing. Indigenous knowledge and local leaders help raise consciousness create knowledge, provide material support to expand referral networks and services, and build commitment within diverse organizations and communities in a context-appropriate and acceptable manner. An external funding agency can play a critical catalytic role by supporting diverse alliances in order to increase access to abortion and other reproductive health services. The state organizers' work suggests that people's ideas and behavior can be changed as well as their relationships with themselves, their organizations, and others. Engaging with people respectfully while fostering different perspectives is crucial to this task.

The US Constitution gives Americans the basic right to decide freely about the number, spacing, and timing of our children -- although it has not yet ensured our right to have the information and means to do so [27]. Right-wing extremists use personal religious beliefs, states-rights ideology, wealth, and interlocking structural systems to trample on the philosophy and spirit of the American Enlightenment. Anti-female beliefs and behavior are maintained as false norms in conservative states. This ideology and practice is inextricably connected to the distribution of power and wealth in our society.

### We are at a crossroads

If the structural and symbolic violence against women's right to health is exacerbated, some of our states could slip further into an abyss. This paper has described a multi-level, integrated, locally-designed and implemented initiative that was operationalized in a number of U.S. states. Supported and strengthened by outside funds, resources and expertise, the project enabled interventions that expanded access to reproductive health services, including abortion. The approach and interventions present an extraordinary opportunity to begin to reverse the inequalities growing over the past years. Power and empowerment are at their core. Organizers have stimulated behavior change, organizational change, and created many new alliances. Hopefully, these efforts can lead to broader collective action to bring about systemic change such that all women will gain what is truly meant by freedom and human rights

## Competing interests

The authors declare that they have no competing interests.

## References

[B1] Hedges C The Rise of the Religious Right in the Republican Party 2004

[B2] Kumashiro KK The Seduction of Common Sense: How the Right Has Framed the Debate on America's Schools 2008 New York and London: Teachers College Press, Columbia University 11

[B3] Kumashiro KK The Seduction of Common Sense: How the Right Has Framed the Debate on America's Schools 2008 New York and London: Teachers College Press, Columbia University 13 14

[B4] Genocide 2010: A Night for Life The Tulsa Times: In Brief: April 24, 2010. http://www.tulsaworld.com/default.aspx. Tulsa, Oklahoma. December 29, 2010. http://www.tulsaworld.com/news/article.aspx?subjectid=11&articleid=20100417_18_A11_hMabee152137&archive=yes

[B5] Kumashiro KK The Seduction of Common Sense: How the Right Has Framed the Debate on America's Schools 2008 15 New York and London: Teachers College Press, Columbia University 48

[B6] MergerWatch No Strings Attached: Public Funding of Religiously-Sponsored Hospitals in the US 2002

[B7] NARAL ProchoiceAmerica. Oklahoma December 29, 2010. http://www.naral.org/government-and-you/state-governments/state-profiles/did-you-know/oklahoma.html

[B8] NewYorkTimes.com James C McKinley Jr Strict Abortion Measures Enacted in Oklahoma 2010 page A14 of the New York edition. December 29, 2010. www.nytimes.com/2010/04/28/us/28abortion.html

[B9] NARALProchoiceAmerica The Truth about Crisis Pregnancy Centers December 29, 2010. http://www.prochoiceamerica.org/media/fact-sheets/abortion-cpcs.pdf

[B10] NewYorkTimes.com James C McKinley Jr Strict Abortion Measures Enacted in Oklahoma 2010 page A14 of the New York edition. www.nytimes.com/2010/04/28/us/28abortion.html

[B11] NARAL ProchoiceAmerica: Health Care Law Holds Promise for Women's Reproductive Health Care 2010 www.naral.org/http://www.prochoiceamerica.org/media/fact-sheets/birth-control-healthy-pregnancies-affordable-care-act.pdf

[B12] Mathematica Policy Research, Inc Impacts of Four Title V, Section 510 Abstinence Education Programs. Final Report 2007 Princeton, New Jersey

[B13] Waxman H The Content of Federally Funded Abstinence-Only Education Part I 2004

[B14] Hedges C Is America Yearning for Fascism? 2010 http://www.truthdig.com/report/item/is_america_yearing_for20683233

[B15] Kumashiro KK The Seduction of Common Sense: How the Right Has Framed the Debate on America's Schools 2008 New York and London: Teachers College Press, Columbia University 11

[B16] Artz L Ortega Murphy B Cultural Hegemony in the United States Foundations of Popular Culture 2000 7 Thousand Oaks: Sage Publications, Inc

[B17] Acker J Feree MM, Martin PY Feminist goals and organizing practices Feminist organizations: Harvest of the new woman's movement 1995 Philadelphia, PA.: Temple University Press 137 144

[B18] Chinn PL Peace and Power: Building communities for the future 2001 Toronto, ON: Jones and Bartlett Publishers

[B19] Freire P Pedagogy for the Oppressed 1968 New York: Herder and Herder **Education for a Critical Consciousness**. New York: Seabury Press. 1973

[B20] Gramsci A Selections from the Prison Notebooks 1971 New York: International Publishers

[B21] Artz L Ortega Murphy B Cultural Hegemony in the United States Foundations of Popular Culture 2000 7 Thousand Oaks: Sage Publications, Inc

[B22] Pratkanis AR Turner ME Persuasion and Democracy: Strategies for Increasing Deliberative Participation and Enacting Social Change Journal of Social Issues 1996 52 1 Spring 187 206 10.1111/j.1540-4560.1996.tb01369.x

[B23] Alinsky S Rules for Radicals 1971 New York: Vintage Books

[B24] Mondros JB Wilson SM Organizing for Power and Empowerment 1994 Columbia University Press. New York

[B25] Mondros JB Wilson SM Organizing for Power and Empowerment 1994 Columbia University Press. New York 250

[B26] Kumashiro KK The Seduction of Common Sense: How the Right Has Framed the Debate on America's Schools 2008 New York and London: Teachers College Press, Columbia University 95

[B27] Grabiner G Lawler J Contemporary Significance of an Article by Mitchell Franklin on Two Earlier Wars on Terror Nature, Society, and Thought 2004 16 4 389 96

